# Smart Polymeric Wound Dressings for Wound Treatment: Contributions and Applications

**DOI:** 10.3390/ijms27167343

**Published:** 2026-08-17

**Authors:** Eduard-Gabriel Constantin, Mădălina Georgiana Albu Kaya, Cristina-Elena Dinu-Pîrvu, Lăcrămioara Popa, Valentina Anuța, Răzvan Mihai Prisada, Mihaela Violeta Ghica

**Affiliations:** 1 Department of Physical and Colloidal Chemistry, Faculty of Pharmacy, “Carol Davila” University of Medicine and Pharmacy, 6 Traian Vuia Str., 020956 Bucharest, Romania; eduard-gabriel.constantin@drd.umfcd.ro (E.-G.C.); lacramioara.popa@umfcd.ro (L.P.); valentina.anuta@umfcd.ro (V.A.); razvan.prisada@umfcd.ro (R.M.P.); mihaela.ghica@umfcd.ro (M.V.G.); 2 Innovative Therapeutic Structures Research and Development Center (InnoTher), “Carol Davila” University of Medicine and Pharmacy, 6 Traian Vuia Str., 020956 Bucharest, Romania; 3 Department of Collagen, Division of Leather and Footwear Research Institute, National Research and Development Institute for Textiles and Leather, 93 Ion Minulescu Str., 031215 Bucharest, Romania; albu_mada@yahoo.com

**Keywords:** smart polymeric wound dressings, wound treatment, theragnostic, drug delivery

## Abstract

Wound management continues to represent a major global healthcare challenge, with the wound care market growing each year and a rising incidence of chronic wounds worldwide. Effective wound healing requires dressings that protect injured tissue, prevent infection, and actively modulate the wound microenvironment to promote tissue regeneration. In recent years, smart polymeric wound dressings have emerged as a functional, more advanced class of wound dressings, engineered from materials capable of responding to stimuli. Physically responsive systems include moisture-adaptive dressings that prevent wound dryness or maceration, pressure-sensitive dressings incorporating flexible capacitive sensors for high mechanical stress mapping, thermoresponsive dressings exploiting sol–gel transitions for temperature-controlled drug release, light-responsive dressings enabling photothermal and photodynamic therapy, and electro-responsive dressings integrating conductive polymers for self-powered electrical stimulation or closed-loop wound monitoring. Chemically responsive systems exploit endogenous biochemical signals, including pH shifts for wound monitoring, reactive oxygen species-cleavable bonds for on-demand drug release, and glucose-responsive platforms for autonomous glycemic regulation in diabetic wounds. Biologically responsive dressings use enzymatic triggers, such as matrix metalloproteinases, hyaluronidase, and bacterial proteases, to achieve autonomous drug delivery. Film-forming sprays further expand the versatility of smart polymeric dressings by enabling contactless application adaptable to irregular wound shapes. In this review, we summarize recent advances in the design, stimuli-responsive mechanisms, characterization methods, and therapeutic outcomes of smart polymeric dressings for wound treatment. Despite promising preclinical results, challenges related to clinical translation, regulatory standardization, and scalable production remain and must be addressed to facilitate widespread clinical adoption. Future directions include multi-stimuli responsive platforms, artificial intelligence-guided wound monitoring, bioprinting of specific dressings, and environmentally sustainable biomaterial design.

## 1. Introduction

Skin is the largest organ of the human body when we are considering either its area or mass. It serves as a primary protective cover, possessing a complex, multilayered tissue architecture [1]. The role of the skin as an external barrier is defined by shielding internal organs from mechanical injury, exposure to radiation, harmful chemical agents and pathogenic agents, such as bacteria and viruses [2,3,4]. Beyond this critical role, the skin also functions as a sensory organ, housing thermoreceptors, nociceptors, pruriceptors, and mechanoreceptors, while it plays a critical role in maintaining body homeostasis through toxin elimination, regulating hydration, preventing electrolyte imbalance, and contributing to the control of body temperature and blood pressure [5,6,7]. Loss of these essential functions has consequences that extend beyond localized tissue damage [1].

Any disruption in the integrity of the skin, mucous membranes, or organ tissue may be defined as a wound. Such structural damage initiates a cascade of biological responses aimed at restoring tissue continuity and function [8,9]. According to Tudoroiu et al., different criteria are used for wound classification: etiology, healing time, depth of injury or number of skin layers affected, complexity, contamination and postoperative infection risk, mode of lesion, tissue loss, appearance, and injured tissue coloration [9]. From those criteria, the most used in clinical practice are etiology (open versus closed wounds) and healing time (acute versus chronic wounds) [10].

Wound healing was described by Guo and DiPietro as a complex dynamic process, consisting of an orchestrated sequence of events involving biologic and immunologic systems, making it a complex biochemical phenomenon that encompasses phases of hemostasis, inflammation, proliferation and remodeling. Those events are continuous, overlapping and precisely coordinated [2,11]. Any interruption or prolongation in the process may lead to delayed or ineffective wound healing, or an absence of wound healing [11,12]. In addition, the progression and efficiency of wound repair are not solely determined by the extent of the injury itself. Rather, wound healing requires a favorable combination of local (oxygenation, infection, wound capillarization, etc.) and systemic (age, gender, stress, overall health, etc.) factors that modulate cellular activity, inflammatory response, and tissue regeneration [8]. The management of the wound-healing process presents multiple healthcare challenges as the incidence of hard-to-heal wounds is rising, affecting the quality of life worldwide [13,14].

The main challenge for wound healing is the permanent infection risk. In an infected state, bacterial biofilm develops and grows, threatening to lead to sepsis. This is doubled by the spread of antibiotic-resistant strains, compromising therapeutic efficacy, especially in chronic wounds [4,15]. A good example is diabetic ulcers, where infection is reported for around 55% of patients. Healing is slowed by inflammatory cytokines, accumulation of reactive oxygen species and poor local blood flow. Wound exudate is also an important factor to be taken into consideration, as it creates a favorable environment for bacterial colonization and may cause the maceration of nearby healthy tissue [16,17]. The global wound care market was valued at over 23 billion dollars in 2024 and is expected to experience a compound growth of 4.19% per year from 2025 through 2030. The market growth is primarily due to rapid technological progress relating to the biomaterials used in wound care [16,18,19].

Across wound types, burns are responsible for over 180,000 annual deaths, according to the World Health Organization (WHO). The main causes of morbidity are burns that are treated either incorrectly or not treated at all. The management of burns requires not only immediate burn protection but also long-term strategies that promote healing and prevent infection [20]. It is important to note that the majority of burn injuries occur in low- and middle-income countries, while the rate of child deaths from burns is currently seven times lower in high-income countries. Hospitalization and immediate treatment for burns vary by country and are heavily influenced by access to medicine, technology and the patient’s socioeconomic status [21,22]. The direct cost of burn care may vary by the severity and area of the burn, but a mean total cost per burn patient in the United States of America is $88,218 (ranging from $704 to $717,306, with a median of $44,024), without including costs like inability to work, emotional stress and family resources (caring for the patient during recovery) [22,23,24].

## 2. Methods

This narrative review was conducted to provide a comprehensive overview of recent advances in smart polymeric wound dressings for the treatment of skin wounds. A literature search was performed in PubMed, Scopus, Springer, ACS, RCS, Web of Science, ScienceDirect, Wiley Online Library publisher and other open-access platforms, and by manual screening of the reference lists of relevant articles. Publications indexed from January 2010 to July 2026 were predominantly considered, with earlier landmark studies included where they were foundational to the field (for example, the original description of moist wound healing).

The search combined the following terms using Boolean operators: (smart OR intelligent OR stimuli responsive OR stimulus responsive) AND (wound dressing OR wound healing OR polymeric film OR hydrogel OR electrospun membrane OR nanofiber OR film-forming spray OR bioelectronic dressing AND drug delivery OR wound monitoring OR biosensor). Keyword combinations were adapted to the syntax of each database.

For the purposes of this review, a smart polymeric wound dressing was defined as a wound contact system, using polymeric components that either (i) alter a therapeutic output, such as drug release, mechanical behavior or antimicrobial activity, in response to a defined endogenous or exogenous stimulus, or (ii) report a wound relevant parameter, such as pH, temperature, moisture or bacterial activity, to the user or to an external device. This functional definition was applied irrespective of the physical format of the dressing. Accordingly, all polymeric systems were eligible for inclusion. This scope was adopted because the responsive chemistries under discussion are, in most cases, transferable between these formats, and because many of the systems reported in the recent literature are hybrids that cannot be assigned to a single format.

Original research articles, review articles and selected landmark publications published in English were eligible. Records were excluded if the dressing contained no polymeric component, if the responsive or sensing function was absent or not characterized, or if only a conference abstract or non-peer-reviewed preprint was available. Titles and abstracts were screened for relevance, and the full texts of eligible records were assessed by the authors, with disagreements resolved by discussion. Studies were selected based on their relevance to the development, design, mechanism of action, physicochemical characterization and therapeutic application of smart polymeric wound dressings.

## 3. Molecular Aspects of Skin Physiology and Wound Healing

Structurally, the skin consists of three distinct layers: the epidermis, dermis, and hypodermis.

The epidermis constitutes the outermost layer and is avascular, primarily composed of keratinocytes (90% of epidermal cells). They undergo continuous proliferation, differentiation, and terminal cornification, forming the stratum corneum that serves as the primary physical and antimicrobial barrier. Other specialized cell types that can be found in this layer are Langerhans cells (involved in immune responses), Merkel cells (mechanoreceptors) and melanocytes (involved in melanin synthesis) [7,25].

Beneath the epidermis lies the dermis, a layer that is predominantly fibrous in nature due to a high content of elastic fibers and collagen. The principal cellular population of this layer consists of fibroblasts (the cells that synthesize collagen, elastin, and proteoglycans). It is also the origin of hair follicles, sebaceous, and sweat glands. This layer is essential for structural integrity and sensory perception [26,27].

The hypodermis is the deepest and thickest skin layer, located between the dermis and the underlying bones or muscles. It consists mainly of loose connective tissue and elastin, serving key functions such as thermal insulation and acting as a nutritional reserve (by storing fat) through adipocytes. Those cells are also involved in inflammation and tissue repair [28].

Following skin injury, the healing process involves a series of coordinated stages that lead to tissue repair [29].

The first phase is called hemostasis (also known as coagulation) and begins immediately after wound occurrence, with vascular constriction. This is done via the release of vasoconstrictive factors by cell membranes [29]. The reflex vasoconstriction is only temporarily effective, as the mechanism lasts for a few minutes. The damaged endothelium and platelets will stimulate the clotting cascade. The clot consists of fibrin and hyaluronan, forming a provisional matrix for wound healing and also serves as a reservoir of cytokines and growth factors that are released from platelets that adhere to this surface [30,31]. Once bleeding stops, the blood vessels adjacent to the wound site facilitate the migration of pro-inflammatory cytokines and growth factors, releasing cells into the wound (chemotaxis). The main growth factors released are transforming growth factor (TGF), platelet-derived growth factor (PDGF), fibroblast growth factor (FGF) and epidermal growth factor (EGF) [12,32].

After this step, inflammation occurs, characterized by the fast immune response, consisting of local infiltration of neutrophils, macrophages, and lymphocytes [30].

Interleukin (IL)-1, together with tumor necrosis factor (TNF), TGF, platelet factor 4 (PF4), and bacterial products, all stimulate neutrophil infiltration to the injured area. The peak concentration of neutrophils is reached 1–2 days after the wounding occurs. The neutrophils are responsible for clearing the wound site of cellular debris by releasing proteolytic enzymes [32,33].

Monocytes start to infiltrate the area 48 to 72 h after injury and differentiate into macrophages. In the early stages of wound healing, macrophages recruit and activate leukocytes and release cytokines. Towards the end of the inflammatory phase, they phagocytose apoptotic neutrophils, cellular debris and residual matrix components, thereby resolving inflammation and enabling the transition to the proliferative phase. In this way, macrophages coordinate the transition from the inflammatory to the proliferative phase [34,35].

The proliferation phase typically starts around day 4 after the patient was injured. It begins with the arrival of fibroblasts at the wound site. The key stages of this phase are epithelialization, angiogenesis, granulation, tissue formation and collagen deposition. The provisional matrix formed during hemostasis is used as a scaffold for angiogenesis. The newly formed vascular path will supply nutrients to the granulating tissue. Vascular regeneration is stimulated by TNF-α and Vascular Endothelial Growth Factor (VEGF) [12,33,34].

The remodeling phase mainly consists of the deposition of collagen in order to form a well-organized structural network. In granulation tissue, collagen fibers are finer than those found in healthy dermis, while also being aligned parallel to the wound site surface (type III collagen). Those fibers must be further consolidated by fibroblasts [26]. A concentration of nearly 40% of type III collagen present at the wound site decreases to almost 10% when the remodeling phase is complete. This is achieved by fibroblasts through a balanced collagenase synthesis (that will conduct the lysis of old matrix) paired with synthesis of type I collagen (to produce the new matrix) [26,34].

The role of collagen in the wound-healing process is well-established, as it is the major protein component of acute wound connective tissue. The biological role of collagen is predetermined by differences in its biochemical composition. Collagen consists of helicoidal structures, composed of glycine, hydroxyproline and proline [26,36].

The most frequent form of collagen in a healthy individual is type I, present in bones, tendons and skin. Type III collagen is found in skin, fascia and blood vessels. Healthy dermis accounts for 80% of type I collagen and 20% of type III. Collagen synthesis requires lysine hydroxylation, proline hydroxylation and cofactors (iron and vitamin C). A lack of cofactors may result in delayed or incomplete wound healing [12,26].

Although wound healing is driven by molecular and cellular events, these processes occur within a local wound environment that can either support or impair healing. Excess exudate, infection, desiccation, hypoxia, and mechanical stress can disrupt the signaling pathways and cellular activities required for effective healing. This connection between biology and the wound microenvironment provides the rationale for adequate wound dressings [37,38].

## 4. Smart Polymeric Systems for Skin Wound Dressings

A wound dressing is described as a sterile and biocompatible material intended to provide a protective local environment that facilitates tissue repair. It plays a critical role in wound treatment, directly influencing the healing process, patient comfort, infection rate, and overall clinical outcomes. The choice of an appropriate dressing is, therefore, essential in creating a protective environment that supports recovery of the skin [38,39].

Traditional wound dressings, such as cotton, wool, or other types of gauze or patches, provide only exudate absorption and limited passive coverage without offering an adequate environment to actively support faster healing of the tissue [40]. Additionally, frequent dressing changes may disrupt the local wound environment, delaying tissue regeneration and reducing the effectiveness of endogenous healing, while also causing discomfort to patients [41].

Modern dressings are advanced biomaterial systems that actively regulate the wound microenvironment and enhance tissue regeneration, while preventing infection. Unlike classical dressings, modern systems maintain a moist wound environment that promotes healing, leading to enhanced collagen synthesis, angiogenesis and cellular migration [38,39]. The shift in the preferred choice for dressings occurred almost 70 years ago, when it was discovered that the moist environment leads to a shorter healing period, with a decrease in unaesthetic scar formation, thus replacing the traditional “dry” approach [42,43].

The modern dressings are classified into hydrocolloids, hydrogels, sponges, foams, alginates and films [44]. Hydrocolloids contain gel-forming agents that are able to absorb exudate while maintaining moisture and facilitating autolytic debridement. Hydrogels are networks of hydrophilic polymers, capable of retaining large amounts of water and are able to moisturize even dry wounds while also assisting in autolytic debridement [44]. Sponges are dry forms obtained by the lyophilization of hydrogels that are porous, lighter than their wet counterpart and offer good gas exchange, while also maintaining a moist environment when hydrated [45]. Foams are able to be used for low- to heavy-exudate wounds and may be used under compression to regulate their exudate intake (a relaxed foam wound dressing will absorb more exudate compared to a compressed one) [46,47]. Alginates are derived from seaweed and form a moist gel as they absorb liquid, making them ideal for heavily exuding wounds [48]. Films have emerged as innovative materials in wound care, being thin, flexible, usually transparent (allowing wound monitoring), and often biodegradable. They can be used both as primary and secondary wound dressings (to keep the primary wound dressing in place). Recently, smart polymeric dressings have begun to appear, promising a potential future improvement of wound dressings [49,50,51].

Smart polymeric dressings are systems engineered with stimulus-responsive functionalities that enable them to adapt and respond to environmental changes. They can be used for controlled drug delivery, wound protection and enhanced healing, due to their customizable properties, further increasing the capability of dressings in the biomedical field [52,53,54,55].

An ideal wound dressing must be effective on any wound type, with the following attributes being very important: the wound dressing promotes local moisture when applied, supports epidermal cells and promotes tissue regeneration, has adequate oxygen permeability but protects against pathogens (semi-permeable), is sterile, is biocompatible, can absorb wound exudates, and is easy to remove when desired [56,57]. A multidisciplinary approach for cutaneous wound care is, therefore, essential to restore tissue integrity and improve outcomes, with the main objective being achieving functional, structural and aesthetic healing [58,59]. The overarching goal of wound management is to shorten the healing process by preventing infection, reducing inflammation and pain, while also minimizing scar formation [13].

A key factor in the design of smart polymeric systems is the choice of polymeric component(s). Table 1 summarizes commonly used polymers in the development of smart polymeric dressings.

**Figure 1:**
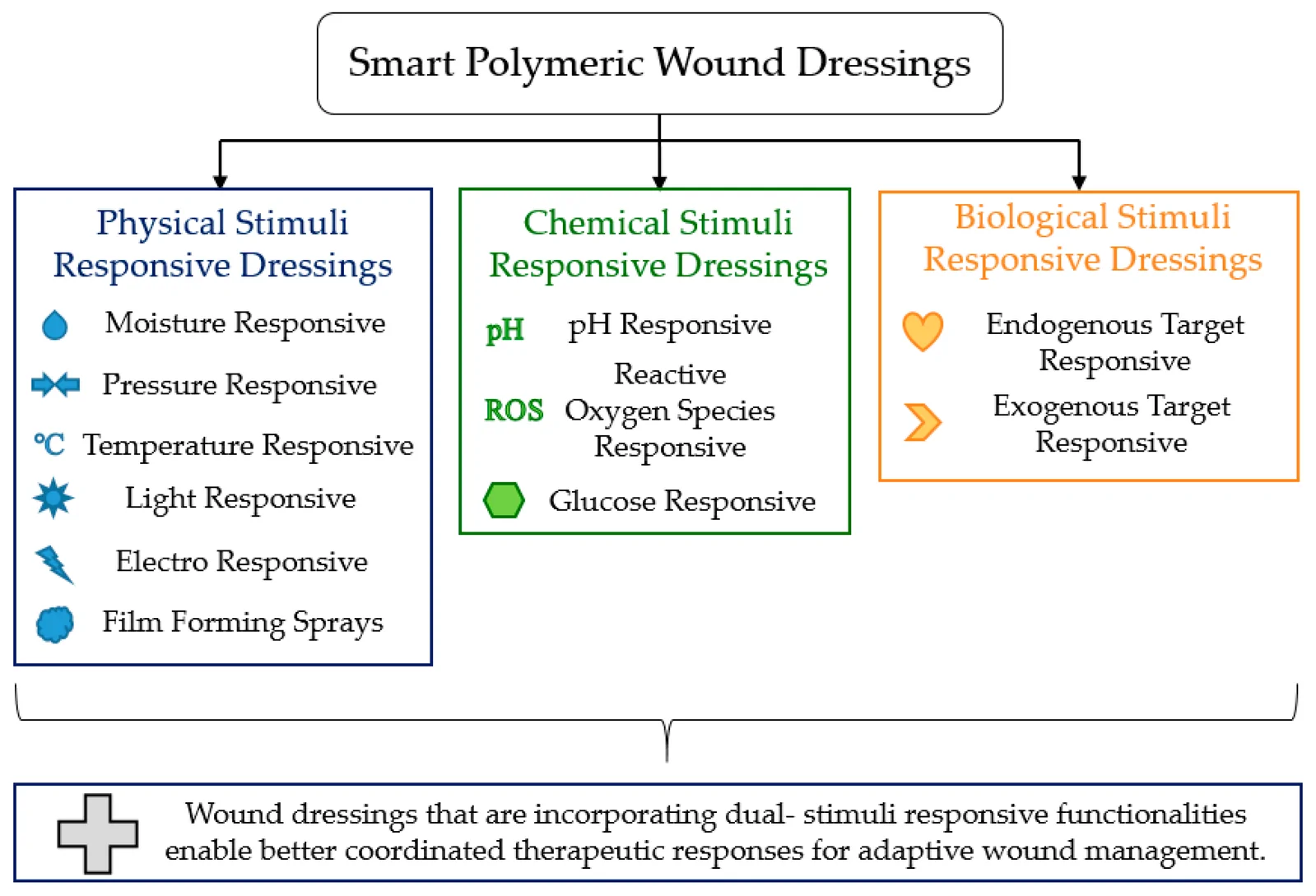
Classification of smart polymeric wound dressings according to stimuli to which they respond.

The following section presents classification criteria for smart polymeric dressings. The criteria were organized according to the principal stimulus to which each system responds, namely physical, chemical or biological. Systems exhibiting more than one responsive mechanism were assigned to the category corresponding to their principal mode of action, with additional functionalities elaborated where relevant. A schematic representation of the proposed classification framework is presented in Figure 1.

### 4.1. Physical Stimuli Responsive Wound Dressings

Physical stimuli-responsive wound dressings represent a class of smart polymeric systems that are capable of structural, mechanical, or functional changes in response to physical forces. In the context of wound-dressing applications, these stimuli primarily include moisture, pressure, temperature, light, electrical fields, and mechanical forces. By using these triggers, they can modulate drug release kinetics, indicate dangerous conditions or adapt to wound site properties. This section reviews the principal types of physically responsive systems developed for wound healing, highlighting their design principles, responsive mechanisms, and therapeutic outcomes [104,105,106].

#### 4.1.1. Moisture Responsive Wound Dressings

Maintaining an optimal level of moisture in the wounded area during all healing phases is crucial. Excessive moisture may result in tissue maceration, while reaching a level of moisture that is too low slows healing and may lead to excessive scarring of the area. The most common causes of high moisture are exudate accumulation from the injury and sweating, while a low level of moisture may be caused by overly frequent dressing changes or a wound dressing with swelling behavior that will absorb excess blood and wound exudates but may cause wound dehydration if the transmission rate is too high. Therefore, it is mandatory for the wound dressing to keep an adequate water vapor transmission rate [13,104,107].

Research indicates that a water vapor transmission rate (WVTR) of approximately 1790–2266 g/m^2^/day is ideal for wound dressings, as this range maintains optimal moisture for epidermal cell and fibroblast proliferation [108].

Currently, the most used moisture-retaining dressings are hydrocolloids. They contain a combination of gelling agents, elastic materials, and adhesives and are usually supported by a film or bandage to enable a proper local effect and limit outside water from destabilizing the hydrocolloid structure [47,109]. By forming a gel layer, hydrocolloid dressings protect the wound and absorb exudate, being efficacious in both chronic and acute wounds. Importantly, patients with infected wounds should not use them, as they are prone to anaerobic infections. People with sensitive skin should be careful with the repeated application and removal of this type of treatment, as it may impact the skin’s stratum corneum [38]. Additionally, hydrocolloid dressings reduce evaporative loss to approximately 20–30% of that from exposed wounds, which may be excessive for some wound types [110]. When balancing between the advantages and disadvantages of hydrocolloid dressings, a modern alternative that should be taken into consideration is represented by polymeric films.

Moisture-adaptive films can be engineered to transition from hydrophobic to hydrophilic, resulting in a “water-triggered expansion” that removes the water between the dressing and tissue, resulting in strong adhesion to the wound site [111]. On the other hand, there are films made from polyethylene oxide and polyethylene glycol-α-cyclodextrin that are capable of “water-triggered shrinkage”. The shrinkage is able to tighten the wound margins, promoting faster closure [112]. The film designed by Yi et al. demonstrated a contraction of approximately 50% within the first 6 s of exposure to water, generating up to 6 MPa of contractile stress [53,112].

The main disadvantages of smart moisture-responsive polymeric films are related to their dependency on ambient humidity, leading to inconsistent or unintended changes to their properties. Additionally, if the moisture response is not properly optimized, the film may lead to the maceration of tissue or formation of ulcers [53].

#### 4.1.2. Pressure Responsive Wound Dressings

Individuals with ulcers and immobile or sedentary patients often undergo pressure relief therapy. Localized pressure leads to blockage of blood circulation, resulting in cell hypoxia and lack of nutritional substances, thus slowing the healing process. Flexible pressure-sensitive dressings are made to monitor wounded areas by converting pressure into detectable electrical signals [113,114].

To fulfill this role, various types of pressure sensors were tested [115]. Among them, each has its own limitations: piezoresistive sensors suffer from signal drift during temperature changes and present high power consumption [116], triboelectric sensors respond only to dynamic pressure changes [117], and thus cannot be advantageously used as static pressure sensors and piezoelectric pressure sensors show hysteresis in response to external pressure (a behavior where output is based not only on the current output, but also on the history of the input, causing inaccurate readings) and electrical crosstalk (electromagnetic interference that cause a signal traveling through a path to affect a nearby path, causing loss of signal integrity and lower accuracy in sensors) [118,119]. Capacitive sensors are usually less impacted by temperature and electrical crosstalk. A flexible capacitive sensor can be built by arranging top and bottom electrodes to form row and column structures, separated by a dielectric layer. The details on how to fabricate this kind of sensor can be found in the paper published by Luo et al. [120]. The sensor fabricated through this method shows a sensitivity of 0.43 kPa^−1^ in a pressure regime < 1 kPa, while also presenting a low pressure detection limit of 4 Pa, with a reaction time of 33 ms.

Different kinds of pressure-responsive smart polymeric systems are the ones that rely on mechanical force to trigger the release of drug molecules entrapped inside them, by physical or chemical methods. Mechanical force can be generated either by compression over their surface or by natural body movements [121].

Various methods of integrating the active substance have been reported, and they can be broadly classified into three models based on the interaction between the polymeric matrix and the drug: (i) the free drug molecules model (the active pharmaceutical ingredient is not bound to the polymeric matrix), (ii) the bound drug model (the drug is associated with the polymeric matrix) and (iii) the multiparticulate model (which combines both free and bound forms) [122,123,124].

**Figure 2:**
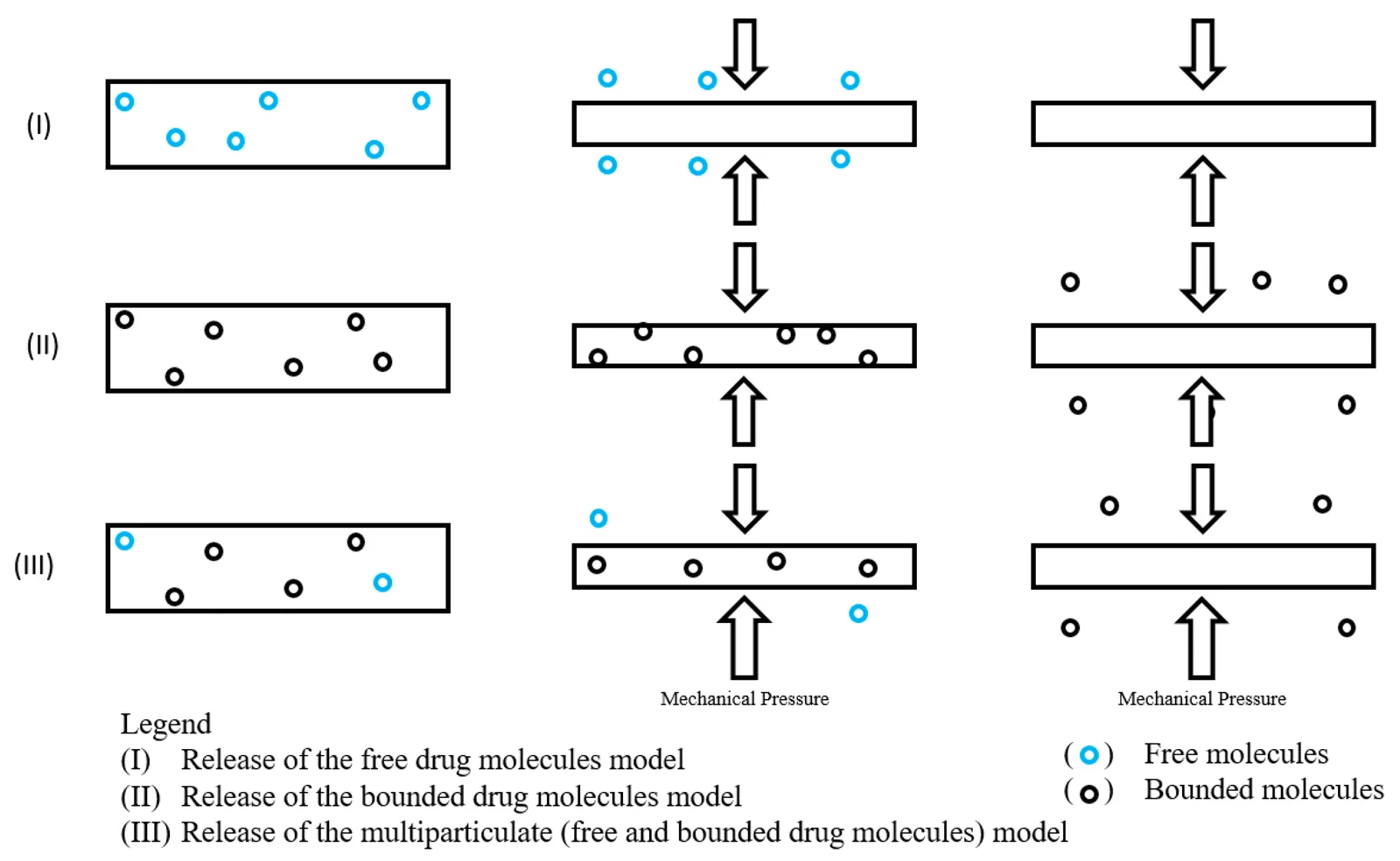
Representation of free, bound and multiparticulate drug molecules release from polymeric wound dressings under mechanical trigger. Adapted from Lee et al. [124].

In the previously mentioned models, drug molecules can be released when pressure is applied, with the main difference being the rate of drug release. In the first model, the drug release is spontaneous. The mechanical force has less impact on the release profile, and drug molecules are quickly exhausted (burst release). In the second model, drug molecules are released over time by dissociation through the polymeric matrix (sustained release). The third model exhibits a biphasic release profile, with an initial rapid release, followed by a prolonged release (controlled release) [124,125,126]. All models are schematically illustrated in Figure 2.

#### 4.1.3. Temperature-Responsive Wound Dressings

During wound healing, a critical parameter that is often neglected is the temperature of the healing wound. The normal temperature of healing wounds should be around 37.8 °C. Deviations over 2.2 °C that are not linked to environmental factors may contribute to tissue deterioration. An increase in wound temperature is also associated with local infection and inflammatory response, while a decrease in wound temperature indicates ischemia, resulting in a slower rate of healing and lower protection from the immune system [13,127,128].

A polymer that exhibits a Lower Critical Solution Temperature (LCST) close to the wound temperature may be used to release drugs, while also adhering to the wound as inflammation is present, as hydrophobic links will result in rapid dehydration of the wound dressing. When LCST is exceeded, as the inflammation disappears, the local temperature will decrease, resulting in a hydrophilic wound dressing that is fit to absorb wound exudate resulting from wound healing. Another advantage of this type of polymer is that a gel may be formed at room temperature, ready to be applied on the skin [88,129]. At skin temperature, as the polymer becomes more hydrophobic, the gel will become a film, fitting the properties needed for liquid wound dressings [130]. Another application is biomaterial ink, which can be used for the 3D printing of thermosensitive hydrogel constructs. The ink was loaded with an antimicrobial component to make the hydrogels produced suitable for wound-healing dressings. Because poly(N-isopropylacrylamide) (PNIPAAm), a polymer with a critical solution point of 32 °C, is used, the final product is able to self-regulate its own swelling ratio and drug diffusion [131]. Chitosan-based hydrogels have also demonstrated a good capability in forming films when heated by body temperature. The downside of this material is the time and financial cost of preparation, which can be overcome over time by innovation [85].

A polymer that presents an Upper Critical Solution Temperature (UCST) above the local wound temperature is less desirable in wound care applications, as it is not suitable to release drugs and form films at skin temperatures [132]. However, polymers that present UCST can be used to address wound exudate management. A network of agar (AG) hydrogel, with a cooling crosslinking effect and UCST characteristics, was prepared and combined with polyethylene glycol (PEG), carboxymethyl cellulose (CMC) and MXene to form a PEG-CMCS/AG/MXene dual network hydrogel that can imitate the function of skin pores. The hydrogel network is destroyed under near-infrared light radiation, showing a photothermal dehydration effect. As the temperature returns to normal, bonds are restored, maintaining a physical barrier that facilitates a moist environment for wound healing. The hydrogel can also be dissolved with L-cysteine methyl ester hydrochloride, avoiding secondary harm during wound-dressing removal. This wound dressing presents a dual responsive capability, as its high conductivity can also promote cell proliferation through electrical stimulation therapy, which is described further in this review [133,134].

Moeinipour et al. developed a multifunctional PVA film with a metronidazole-loaded hydrogen-bonded organic framework and anthocyanin. The film exhibited dual temperature- and pH-responsive drug release, with metronidazole release triggered at temperatures above 37 °C and under alkaline pH conditions. At the same time, the incorporated anthocyanin enabled colorimetric ammonia detection through visible color changes in response to pH fluctuations at the wound site, quantifiable via RGB color detection software [111].

#### 4.1.4. Light Responsive Wound Dressings

Light-responsive systems are active wound dressings that can be enabled through light irradiation, using light as a noninvasive external stimulus. Smart wound dressings of this category function through three primary phototherapeutic modalities: photothermal therapy (PTT), photodynamic therapy (PDT) and photoelectric therapy (PET) [105,106].

PTT converts absorbed infrared (IR) light energy into localized heat, achieving temperatures that kill bacteria through protein denaturation while promoting wound healing through enhanced blood flow and cellular metabolism [105].

A moderate temperature rise (42–50 °C) leads to an overexpression of Heat Shock Proteins (HSPs), while also being correlated with the upregulation of growth factors [135]. This effect is associated with improved repair for host tissue. Within a comparable range (45–55 °C), bacteria enter a state in which their cell membranes are intact, but they cannot proliferate [136].

For short irradiation times, denaturation of host proteins is observed to begin at approximately 50 °C and the mode of cellular death gradually shifts from apoptosis to necrosis at around 58 °C and to necrosis above 61 °C [136]. This creates an obstacle, as temperatures above 58 °C reliably kill bacteria but may injure surrounding viable tissue, while a lower temperature may be insufficient. In a murine wound model, better healing efficacy was reported at 50 °C than at 58 °C. A further complication is that bacterial heat shock proteins may confer thermal tolerance and limit the efficacy of low-temperature PTT [136,137].

The temperatures reported to effectively kill bacteria are in the 50–60 °C range, but this should not be interpreted as a fixed threshold. Thermal injury follows an Arrhenius damage integral, depending on both the temperature and the duration of exposure [136,137].

PDT generates cytotoxic reactive oxygen species (ROS), such as singlet oxygen and radicals, through photosensitizer activation, providing antibacterial effects through oxidative damage of bacteria. The mechanism of PDT may be described as follows. Upon photon absorption, the photosensitizer is promoted from its ground state to an excited singlet state, which crosses by intersystem crossing into a longer-lived triplet state. From the triplet state, two pathways may proceed, with both of them generally happening simultaneously. The type I reaction is characterized by electron transfer with a substrate that generates radical species, and the type II reaction is represented by energy being transferred directly to ground-state molecular oxygen to yield singlet oxygen, generally regarded as the principal cytotoxic species [138,139,140].

The ROS generated are, however, not selective for bacteria. Excess ROS may also damage surrounding host tissue and increase the local inflammatory response, which restricts the applicability of PDT in wounds and is a principal cause of non-healing chronic wounds [141,142].

Complete ROS elimination is also not desirable since low concentrations of ROS act as physiological messengers. Low concentrations of ROS facilitate angiogenesis in wound healing through VEGF and VEGF receptor signaling, while PDGF signaling in wound angiogenesis is dependent on H_2_O_2_ for its biological function [141].

Controlled ROS production has been shown to be the most beneficial [143]. PDT-based dressings should be designed and evaluated with a therapeutic window in mind, rather than maximizing ROS output. Reported strategies include converting high-dose superoxide and hydroxyl radicals into a lower dose of a more reactive species while simultaneously activating host antioxidant pathways, and dual-phase systems that generate bactericidal ROS under irradiation but scavenge excess ROS in the absence of light to relieve oxidative stress in the later, reparative phase [144,145].

PET generates photoelectrons that influence cellular behavior, including migration and proliferation, properties that overlap with electro-responsive wound dressings. While PDT and PTT have been extensively studied, the role of PET in wound healing has only recently gained attention, leaving a gap in mechanistic understanding and clinical translation [106].

A particularly important direction in this field has been the development of sequential strategies that leverage multiple phototherapeutic modalities in a coordinated manner to address the distinct demands of different wound healing phases (with pH-sensitive indicators enabling the optimized timing of interventions) [144,146,147]. This sequential strategy has demonstrated efficacy in anti-infection outcomes, while also reducing wound inflammation and providing a supportive environment for skin and superficial skeletal muscle repair across animal models [105].

The selection of the light source wavelength is a critical design parameter that influences the clinical utility of these systems. Near-infrared light (NIR), with wavelengths ranging between 700 and 1100 nm, is preferred to activate light-responsive wound dressings, as it offers superior tissue penetration (compared to visible light) and minimal phototoxicity (compared to ultraviolet radiation) [146,147,148].

#### 4.1.5. Electro-Responsive Wound Dressings

Electro-responsive systems are wound dressings that use conductive polymers and electroactive materials to deliver exogenous electrical stimulation, amplify endogenous bioelectric signals (accelerating wound healing) or serve as drug delivery and wound monitoring sensors [149,150]. Electro-responsive wound dressings demonstrate efficacy across multiple wound types. This therapeutic effect is possible through multiple molecular pathways. Electrical stimulation creates a directional electric field gradient that guides cellular migration and modulates cytokine expression by reducing inflammatory markers, while increasing growth factors. The most commonly used conductive polymers include polypyrrole, polyaniline and polythiophene [149,151].

The big disadvantage of conductive polymers (CPs) is that they are typically brittle, poorly soluble, and lack the mechanical compliance required for wound dressings when used alone. To overcome these limitations, CPs are commonly incorporated into composite films or hydrogels [152]. For example, Don et al. developed a polypyrrole–ulvan/chitosan (PPy-U/C) film in which PPy nanoparticles (237–323 nm) were synthesized in situ within an ulvan solution to achieve stable aqueous dispersion. The resulting films exhibited adjustable conductivity (10^−2^–10^−3^ S·m^−1^), a water vapor transmission rate of 1400–1640 g·m^−2^·day^−1^, swelling ratios between 255 and 518%, and antibacterial activity against *Staphylococcus aureus*, *Escherichia coli*, and *Pseudomonas aeruginosa*. Under electrical stimulation, the films enhanced fibroblast proliferation, and in vivo studies demonstrated complete wound closure within 14 days [153,154].

Similarly, Zhai et al. reported a tannin cellulose polypyrrole dressing that amplifies endogenous electric fields while providing antibacterial action from tannic acid. In vivo, this dressing accelerated wound closure through enhanced granulation tissue formation and increased angiogenesis [155].

Beyond PPy-based systems, poly(3,4-ethylenedioxythiopene):polystyrene sulfonate (PEDOT:PSS) blends have been incorporated into conductive dressings. Wang et al. developed a PVA/PEDOT:PSS/cyclodextrin–polyoxometalate (CD-POM) dressing that integrates electrical stimulation with reactive oxygen species (ROS) responsiveness. The dual stimuli-responsive functional design addresses both bacterial biofilm formation and oxidative stress [156].

A big disadvantage of electrotherapy is the requirement of an exogenous power source. Self-powered electro-responsive wound dressings are being developed to mitigate the disadvantage of older-generation devices [157].

Piezoelectric wound dressings exploit materials such as polyvinylidene fluoride (PVDF) and poly-L-lactic acid (PLLA) that generate voltage in response to mechanical deformation [158,159]. Wang et al. constructed a self-powered PVA/PVDF system that converts mechanical energy from physical activity into local piezoelectric stimulation. In diabetic rat wound models, this promoted re-epithelialization, collagen deposition, and angiogenesis while polarizing macrophages from the pro-inflammatory M1 to the reparative M2 phenotype [159]. Wu et al. further refined this approach with an MXene-coated PLA nanofibrous membrane that generates piezoelectric voltages that biomimic endogenous bioelectric potentials, significantly accelerating wound closure while suppressing inflammatory responses [160]. This represents a dual-responsive membrane.

Tang et al. assembled a self-powered patch from electrospun polymer triboelectric layers and a polypyrrole-coated electrode. This wound dressing achieved over 96% bacterial killing through the synergistic effects of electrical stimulation and positive surface charges on the PPy electrode. The patch enabled infected diabetic rat wounds to heal within 2 weeks [161].

Another design was reported by Huo et al., who developed a dual-mode dressing that integrates a triboelectric nanogenerator and an Ag/Zn battery on a fabric substrate. This design adapts to the wound. In the early healing stages, wound exudate acts as an electrolyte to activate the Ag/Zn battery for direct current stimulation. As the wound dries, reduced humidity allows for nanogenerator operation for pulsed electrical signals generated through mechanical force. In rat models, this dual-stimuli-responsive adaptive dressing achieved 89.7% wound closure by day 10, significantly outperforming the control group [162].

Shan et al. fabricated an antibacterial chitosan/PPy wound dressing with dual-stimuli (pH and voltage)-responsive ciprofloxacin release. The PPy coating restores endogenous electric fields, while the pH-responsive chitosan matrix enables drug release in the acidic microenvironment of infected wounds, with additional electrically triggered release under applied voltage [163].

The most advanced systems integrate sensing, stimulation, and drug delivery into closed feedback-loop platforms. Jiang et al. developed a wireless, flexible bioelectronic system capable of on-demand adhesion and detachment. The system continuously monitors skin impedance and temperature and delivers electrostimulation in response to the wound environment. Across preclinical wound models, the treatment group healed approximately 25% more rapidly, with an approximately 50% enhancement in dermal remodeling, with confirmed activation of regenerative genes in monocyte and macrophage populations [164].

Despite their promise, electro-responsive polymeric wound dressings face several implementation issues. Long-term stability of conductive polymers in the moist wound environment remains a concern, as does maintaining the balance between high conductivity and adequate mechanical properties [149,151].

Many self-powered systems generate relatively low and variable electrical outputs that may not consistently reach therapeutic thresholds across different wound types and patient activity levels [157,165]. Additionally, the standardization of electrostimulation parameters, including optimal voltage, frequency, waveform, and duration for different wound types and healing stages, remains an unresolved question that limits clinical translation [166,167].

#### 4.1.6. Film-Forming Sprays (FFS)

Film-forming sprays (FFS) are liquid wound-dressing systems that quickly form a membrane on the wound surface when applied. FFSs offer two main advantages over conventional dressings: there is no need for skin contact during application, decreasing the risk of accidental contamination, and the liquid polymer is adaptable to irregular wound geometries.

Both natural and synthetic polymers with in situ film-forming properties can be used to formulate FFS. The choice of polymer, plasticizer, and solvent system determines the film-drying time and mechanical properties.

The integration of stimuli-responsive materials into FFS platforms represents an emerging strategy. Thermoresponsive FFS exploit the sol–gel transition of thermosensitive polymers to achieve in situ film or gel transition upon contact with body temperature. Prabhu et al. developed a thermoresponsive sprayable delivery system for burn and diabetic wound care, using HPMC and Poloxamer enriched with curcumin and Asiatic acid. The optimized thermosensitive gel spray remained a sprayable liquid at room temperature and transitioned to a gel at skin temperature, achieving in vitro release of up to 45% of both active ingredients in 7 h (compared to 30% for control gels) and up to 90% cumulative release after 24 h. In vivo studies in rats demonstrated that the spray significantly promoted epithelialization and vascularization of the wounded area, outperforming silver sulfadiazine and conventional gel formulations [168].

Similarly, Alparslan et al. developed a thermoresponsive sol–gel spray formulation incorporating polyhexamethylene biguanide using Poloxamer as a carrier. The formulation remained a sprayable liquid at room temperature and instantly gelled upon contact with body temperature, achieving antimicrobial efficacy, high biocompatibility and maintaining fibroblast migration capacity, which is crucial for wound regeneration [169].

Lu et al. designed a sprayable film containing nanocellulose and L-serine-modified silver nanoparticles, capable of rapidly forming a transparent porous film on the skin. The nanocellulose created a lightweight porous structure that enhanced air permeability and drug-carrying capacity, while the L-serine modification enhanced antibacterial efficacy through inherent antimicrobial properties and bacterial targeting capability. In vivo, mice treated with this spray film demonstrated superior wound healing, normalization of keratin thickness, increased hair follicle count, and reduced inflammatory markers [170].

Karwa et al. developed and optimized a rutin-loaded FFS using Eudragit^®^ RLPO and Eudragit^®^ E100. The optimized formulation exhibited very fast drying (under one minute), suitable viscosity (3.8 ± 0.076 cPs), and sustained delivery over 8 h. In vivo wound-healing studies in rats showed complete wound closure by day 16, with histopathological examination showing better epithelialization, collagen deposition, and angiogenesis [171].

Patel et al. used simvastatin in a bioactive FFS formulation using chitosan, collagen, and hyaluronic acid, achieving rapid film formation (under four minutes) and sustained drug release. In vivo excision wound models confirmed efficacy in wound contraction, tissue remodeling and re-epithelialization, while downregulating TNF-α and IL-6 [172].

Dual-responsive sprayable systems represent advanced FFS. Rungrod et al. developed a sprayable hydrogel based on thermo- and pH dual-responsive polymers, combining Pluronic F127 and N-succinyl chitosan. The formulation exhibited favorable sprayability, appropriate gelation properties, and controlled drug release, with antioxidant capacity enhanced by incorporation of *Azadirachta indica* extract. Both extract release and antioxidant activity increased in a dose-dependent manner [173].

Despite their advancement in recent years, FFSs face several challenges. The lack of standardization of characterization methods and evaluation parameters creates difficulties for pharmaceutical development and regulatory approval. Drying time, sprayability, and film uniformity are critical parameters that must be carefully optimized, as they are influenced by polymer type, concentration, plasticizer content, and solvent system. Additionally, the balance between rapid film formation and sustained drug release remains a challenge, as faster drying may limit drug diffusion, while slower drying may compromise the protective barrier function. The scalability of FFS production and long-term stability of stimuli-responsive components within the spray formulation also require further investigation.

### 4.2. Chemical Stimuli Responsive Wound Dressings

Chemical stimuli-responsive wound dressings constitute a class of smart polymeric systems that undergo functional changes in response to the biochemical stimuli present in the wound microenvironment. Biochemical signals serve as biomarkers of wound status, enabling chemical stimuli-responsive wound dressings to autonomously detect and respond to changes during wound healing without external intervention. By using these triggers, they can achieve autonomous drug dosage and dynamic modulation of the healing cascade, offering a feedback-loop therapeutic strategy designed for the evolving needs of a wound [13,54,70]. This section reviews the principal categories of chemically responsive polymeric dressings developed for wound healing: pH-, ROS- and glucose-responsive systems.

#### 4.2.1. pH-Responsive Wound Dressings

pH is a factor that may be used to determine the condition of healthy and unhealthy skin. Healthy skin has a surface pH between 4.5 and 6.5. Immediately after tissue injury and after bacterial colonies develop on the wounded surface, the local pH will become basic (between seven and nine) [174,175].

Among the crosslinking strategies applicable to smart wound dressings, dialdehyde crosslinking offers a dual advantage: biocompatibility and intrinsic stimulus responsiveness. Dialdehyde derivatives of polysaccharides, such as 2,3-dialdehyde cellulose (DAC) and dialdehyde starch (DAS), are obtained through periodate oxidation, generating aldehyde groups that form Schiff base bonds with amine-containing polymers like chitosan or proteins. Unlike glutaraldehyde, these crosslinkers are derived from the polysaccharide matrix itself and carry lower cytotoxicity concerns. Critically for smart dressing design, Schiff base linkages are pH-reversible. They remain stable under neutral to mildly acidic conditions but hydrolyze as pH increases, making them candidates for pH-triggered drug release in infected wounds. The growing availability of dialdehyde polysaccharide crosslinkers provides a versatile platform for engineering responsive networks into smart wound dressings [176,177,178,179].

The different methods developed to track local wound pH are electrochemical, colorimetric, and fluorimetric [174,180,181].

Electrochemical methods imply the use of small electrodes to measure currents. This method is particularly effective because it offers quantitative and real-time measurements. However, there are several disadvantages: difficulty in miniaturizing potentiostats, the need for an external power source, and the need for anti-fouling electrodes, as fouling electrodes will stop working in prolonged contact with the wound fluids or may indicate false pH values, leading to a false diagnosis [181,182,183]. Zhang et al. addressed the antifouling issue in wearable potentiometric wound sensors, noting that antifouling abilities in wound fluids have “rarely been investigated”. They developed a zwitterionic hydrogel coating to suppress the adsorption of bacteria, proteins, and cells, demonstrating that without coating, electrode fouling significantly compromises sensor reliability [182].

Sharp details the surface deposition of 1,2-diaminobenzene to prevent biofouling on printed composite electrodes for wound pH monitoring, confirming that biofouling is a recognized limitation requiring specific countermeasures. This requires repeated changes of electrodes over the healing period [184].

Two additional sources of error are frequently reported in wound sensor studies. The first one is potential drift, which comes both from the reference electrode and from the liquid layer and ion exchange processes at the contact interface of the ion-selective surface. Reported drift magnitudes vary depending on the electrode architecture. For example, solid contact ion-selective electrodes drift at approximately 0.21 ± 0.05 mV/h. Mao et al. designed a sensor with very low potential drift (0.06 ± 0.03 mV/h) compared to a classical one [185,186].

The second additional source of error is selectivity in the protein and electrolyte of wound exudate. Potentiometric selectivity should be expressed as formally determined selectivity coefficients using the separate solution or fixed interference methods, since incorrectly determined coefficients do not predict real sample behavior. In clinical practice, protein and lipid content, altered water displacement and hydrophobic interferents are established causes of systematic error [186,187].

Mitigation strategies for signal drift may be classified into three categories: membrane and interface engineering (primary ion preconditioning, diffusion-limiting polymers, electrical shunting) that suppresses drift at the source and can eliminate user end conditioning and recalibration; differential configurations in which a reference transducer cancels common-mode contributions [187,188]; and ratiometric sensing with selective permeability layers, self-healing electrodes, and chemometric or machine learning approaches for drift compensation and adaptive calibration [189]. Where none of these approaches is implemented, repeated electrode replacement over the healing period remains necessary.

Among wound devices, the textile pH sensor of Mariani et al. shows a reversible response of 59 ± 4 μA per pH unit over pH 6–9 that was not substantially affected by common chemical interferents or by temperature between 22 and 40 °C, and was tested in flow with synthetic wound exudate [181].

Colorimetric methods rely on the incorporation of pH-sensitive dyes into the wound-dressing material, through microbeads or fibers. Examples of natural pH-sensitive dyes are anthocyanins [190], shikonin [191], alizarin [192] and curcumin [193]. Examples of synthetic pH-sensitive dyes include phenol red [70,89], bromothymol blue [194], bromocresol green [195], methyl red and rose Bengal [196]. The dyes change color based on pH conditions, providing both naked-eye estimations and software-based analysis. Methods for software-based analysis require electronic devices ranging from optoelectronic probes to a normal smartphone camera. In some examples, a single picture of a pH-responsive film is taken, and by using a specially designed algorithm, the color is associated with a value on the pH scale. This method is advantageous, as many people own a smartphone and may be able to use the software for performing self-diagnosis, saving time and money [70,197,198].

Fluorimetric methods involve the use of ultraviolet light to produce the electronic excitation of an analyte formed in the interaction between the wound and the wound-dressing. At low concentrations of the analyte, the intensity of the emitted light is directly proportional to the concentration of the analyte, allowing this method to be used for analyte quantification. The downside of this method is represented by the need for UV lamps and fluorescence spectrophotometers [180,199].

Colorimetric and fluorimetric methods can also be combined for more accurate pH monitoring [180]. These methods increase the price of the final product but may be used as a real-time emergency signal for a wound that will become chronic or has been infected [200].

#### 4.2.2. Reactive Oxygen Species (ROS) and Glucose Responsive Wound Dressings

Reactive oxygen species (ROS), including superoxide, hydrogen peroxide (H_2_O_2_), free hydroxyl radicals (-OH), and hypochlorous acid (HOCl), are critical mediators of wound healing. At low physiological concentrations, ROS serve essential functions in antimicrobial defense, platelet activation, cell signaling, and angiogenesis [141]. However, in chronic wounds such as diabetic foot ulcers, venous leg ulcers, and pressure injuries, excessive ROS accumulation creates a self-perpetuating cycle of oxidative stress, sustained macrophage polarization, mitochondrial dysfunction, and extracellular matrix degradation. This prolongs the wound inflammatory phase [201,202]. Redox analysis of wound fluids has confirmed that chronic wounds exhibit significantly higher levels of VEGF, IL-8, and TNF-α compared to acute wounds, reflecting this pathological oxidative imbalance [203].

This dual aspect of ROS (beneficial at physiological levels but dangerous in excess) provides both a therapeutic target and an endogenous trigger for smart dressings [141,204].

Notably, the diabetic wound microenvironment is characterized not only by elevated ROS but also by persistent hyperglycemia, which further increases oxidative stress [142]. Both ROS and glucose are pathologically elevated and interconnected in diabetic chronic wounds and share a common molecular-sensing platform consisting of phenylboronic acid. ROS-responsive and glucose-responsive systems are closely related and are, therefore, discussed together in this section. Local hyperglycemia is a primary driver of oxidative stress in chronic diabetic lesions [205,206].

ROS-responsive polymeric wound dressings are designed to exploit the oxidative microenvironment of chronic wounds, enabling on-demand drug release and dynamic modulation through chemical bonds that selectively cleave in the presence of pathological ROS concentrations or glucose [207].

Phenylboronic acid (PBA)-based boronate ester bonds represent the most extensively investigated ROS-cleavable system in wound-dressing design. PBA reacts with diol-containing polymers like polyvinyl alcohol (PVA), polysaccharides, or gelatin to form dynamic boronate ester crosslinks that undergo oxidative cleavage in the presence of H_2_O_2_ at concentrations similar to those of chronic wounds [205,207].

The oxidative cleavage triggered by ROS induces a controlled degradation of the polymeric network, facilitating the controlled delivery of entrapped therapeutic agents. At the same time, the oxidation reaction of this mechanism consumes the ROS, basically modulating oxidative stress directly at the wound site. Such characteristics enhance the therapeutic potential of the biomaterial in complex tissue restoration and remodeling processes [207,208].

A technology for glucose-responsive wound dressings consists of an autonomous system composed of Au-MoS_2_-PBA nanozymes (nanomaterials with enzyme-like behavior) integrated with hypoxia-sensible microcapsules. In pathological hyperglycemic conditions, the nanozyme utilizes glucose as a substrate to generate ROS and releases insulin for glycemic regulation over a 12 h duration. To prevent hypoglycemia, when homeostatic values are achieved, the activity shifts to supply molecular oxygen, which inhibits further insulin delivery and prevents the onset of hypoglycemia. This type of system has been observed to accelerate tissue restoration rates by approximately three times compared to standard dressings [209].

Other platforms, such as the OAC-10BH hydrogel, use borate ester. In these systems, elevated glucose levels induce bond cleavage to facilitate the localized release of antiinflammatory and antimicrobial agents like berberine. Once glucose concentrations normalize, the borate ester linkages reform, effectively stopping the drug delivery process [210].

Clinical data from human randomized trials, such as the study conducted by Sandroni et al., demonstrate that ROS-responsive delivery systems exhibit superior therapeutic efficacy, achieving a 60.9% reduction in the surface area of chronic wounds compared to a 38.7% reduction observed with conventional dressings [211]. However, the successful clinical translation of these advanced polymeric scaffolds is currently constrained by significant barriers, including the in vivo heterogeneity of reactive oxygen species, the necessity for safety evaluations of degradation byproducts and regulatory standardization. Recent reviews indicate that ROS-responsive systems constitute a promising therapeutic strategy. Future advancements must prioritize the optimization of these systems to ensure a viable balance between performance and feasibility [212,213,214].

### 4.3. Biological Stimuli-Responsive Wound Dressings

Biological stimuli-responsive wound dressings represent a class of smart polymeric wound dressings that undergo functional changes in response to the biological markers generated by activities of cells. Both the host and microorganisms present at the wound site generate enzymes that can be used by incorporating substrates or linkages that are selectively cleaved by the biological agents. This section reviews the principal categories of biologically responsive systems developed for wound healing, organized into endogenous and exogenous target-responsive wound dressings [54,215].

#### 4.3.1. Endogenous Target-Responsive Wound Dressings

Enzymatic activity varies significantly between acute and chronic wounds. Among the most relevant enzymes for enzyme-responsive dressings are matrix metalloproteinases (MMPs), a family of endoproteases responsible for extracellular matrix (ECM) metabolism [216,217].

In chronic wounds, the effect of MMPs and their improper regulation by tissue inhibitors of metalloproteinases (TIMPs) creates a proteolytic imbalance that degrades growth factors, receptors, and ECM components. This results in a sustained inflammatory state and delayed wound healing [218]. This enzymatic mechanism provides the biological explanation for enzyme-responsive polymeric wound dressings. By incorporating bonds that are selectively cleaved by wound enzymes, these systems are designed to release drugs preferentially under protease action. This is a correlative relation and not a proportional one [54,55]. Moreover, the release from an enzyme-cleavable matrix is not determined by enzyme concentration alone, but also by drug diffusion, crosslink density, network architecture, swelling behavior, accessibility of the cleavable substrate to a macromolecular enzyme, local pH and temperature [219,220]. It is worth noting that the majority of MMP-responsive systems in the current literature are formulated as hydrogels. Multiple design strategies have been used over time.

Varshosaz et al. fabricated an electrospun nanofibrous membrane based on modified polybutylene adipate–terephthalate/gelatin loaded with doxycycline, an established MMP inhibitor. The membrane released approximately 65% of doxycycline over 20 h, inhibited MMP activity (confirmed through gel zymography) and improved wound closure, re-epithelialization, collagen deposition, and angiogenesis in rat models [221].

Another commonly used material for MMP-responsive wound dressings is gelatin, because, as a denatured collagen derivative, it is inherently susceptible to MMP proteolysis [222]. Ionescu et al. developed systems constituted of modified gelatin, developed via crosslinking. These films exhibited proper wound-healing properties and outperformed the commercial dressing used as a control, with faster progression through healing phases (inflammation to proliferation and contraction). Since gelatin is inherently susceptible to MMP proteolysis, these films are degraded by wound MMPs, though the study focused on healing outcomes rather than MMP-triggered drug release specifically [223].

Bilgiseven et al. designed a multilayer electrospun nanofiber wound dressing (PU/PEG-quercetin/PVA/gelatin) and specifically analyzed MMP-9 mRNA expression in wound tissue, demonstrating that the nanofiber dressing modulated MMP-9 levels during healing. Complete wound closure was observed after 48 h in vitro, and accelerated healing was confirmed in vivo [224].

Protein nanostructures offer a strategy for enhancing both the responsiveness and biological performance of smart wound dressings. Proteins such as whey protein, silk fibroin, and spirulina protein can be processed into nanoparticles or nanogels. The enzymatic degradability of protein nanostructures by wound proteases (such as MMPs) positions them as inherently responsive components. The protein nanophase can degrade in response to protease activity in chronic or infected wounds, releasing the encapsulated therapeutic agent [101,225,226].

Hyaluronidase, an enzyme that degrades hyaluronic acid (HA), is also upregulated in infected and inflamed wound environments. HA-based systems can, therefore, be engineered for enzyme-dependent biodegradation, enabling infection-triggered drug release [227]. Guan et al. developed an in situ forming film through covalent bond crosslinking. The system demonstrated dual pH- and hyaluronidase-dependent responsiveness. This is achieved by cleaving the HA network, releasing increased amounts of sisomicin sulfate for sustained antibacterial activity. In a mouse skin model, the dressing increased wound healing, as confirmed by histological examination [228].

Systems based on glucose oxidase (GOx) achieve glucose responsiveness through enzymatic activity by catalyzing the oxidation of wound glucose into gluconic acid and hydrogen peroxide (H_2_O_2_). The usage of glucose by GOx directly lowers the local hyperglycemia, addressing the main cause of delayed healing. Additionally, the production of gluconic acid induces a pH reduction, which can serve as a trigger for a pH-responsive drug delivery mechanism of a wound dressing [229,230]. Tang et al. designed a glucose-activated wound dressing combining an electrospun PCL nanofiber membrane layer with a 3D hydrophilic sponge. GOx was grafted onto metal–organic frameworks and embedded in the PCL membrane via ligand interaction. GOx consumes glucose while modulating pH, activating peroxidase-like activity of the framework to generate -OH. In vivo, the dressing achieved a 54% healing rate on day 3 against antibiotic-resistant infected diabetic wounds [231].

Jankowska et al. designed a fluorescent sensing system based on GOx and horseradish peroxidase immobilized on a biocompatible polysaccharide matrix, enabling the simultaneous detection of pH and glucose concentration for wound monitoring, and being able to distinguish between healing and chronic wounds at an early stage [232].

#### 4.3.2. Exogenous Target-Responsive Wound Dressings

Beyond endogenous enzymes, bacterial proteases secreted during wound colonization and biofilm formation provide triggers for responsive wound dressings [233].

Enzyme-responsive polymeric systems for wound healing offer the significant advantage of autonomous drug delivery without requiring external stimuli. However, several challenges remain. The variability of enzyme concentrations across wound types, patient populations, and healing stages necessitates careful calibration of substrate sensitivity and degradation kinetics for each patient. Additionally, the potential for degradation by nonpathological enzyme activity must be addressed [54,218].

Currie et al. developed a highly sensitive bacteria detection method using an electrospun nanofibrous polyurethane membrane incorporating a hemicyanine chromogenic part with an ester linkage that is enzymatically cleaved by bacterial lipase. The membrane achieved a five-times faster chromogenic response than conventional nanofibers, enabling detection at clinically relevant bacterial concentrations within 2 h [96].

Abdali et al. designed bacteria-responsive nanofibrous membranes based on a blend of polycaprolactone/poly(ethylene succinate) that degrade in the presence of bacteria through both lipase secretion and acidic pH, releasing the incorporated biocide. Core–shell nanofibers provided controlled, on-demand release with >1 log bacterial reduction within 2 h and minimal cytotoxicity to human fibroblasts [234].

Most current systems remain in preclinical evaluation, and standardized in vivo models and clinical testing represent the next steps to advance these platforms toward daily clinical practice.

## 5. Characterization Methods for Smart Polymeric Wound Dressings

The characterization of smart polymeric wound dressings is critical for understanding their structural, physicochemical and functional properties. Those properties directly impact their application in wound dressings. A variety of methods are used to ensure that dressings are thoroughly characterized [235,236,237].

A list of characterization methods for smart polymeric wound dressings and their relevance can be found in Table 2.

For the particular case of drug-releasing smart wound dressings, reporting cumulative drug release curves alone is insufficient to distinguish stimulus-triggered release from passive diffusion. Kinetic modeling can provide additional information on the release mechanism and should be considered an important component of characterization. Commonly used models include zero-order, first-order, Higuchi, and Korsmeyer–Peppas models, with the corresponding coefficient of determination (R^2^) and the release exponent (n) reported [70,122,269].

The zero-order model describes a constant release rate over time, such that the amount of drug released increases linearly with time. A good fit to this model may indicate relatively controlled and sustained release, in which the release rate remains approximately independent of the amount of drug remaining in the matrix. This behavior is desirable for systems designed to maintain a constant drug release [270,271].

The first-order model assumes that the release rate is related to the amount of drug remaining in the delivery system, resulting in a progressively decreasing release rate as the drug is depleted. This model is useful for describing systems in which release is faster at the beginning and gradually decreases over time [272,273].

The Higuchi model describes drug release primarily as a diffusion-controlled process from a matrix, with the cumulative amount of drug released proportional to the square root of time [272,274].

The Korsmeyer–Peppas model is particularly useful for describing the contribution of diffusion and polymer relaxation to drug transport. For planar systems, an *n* value of approximately 0.5 is generally associated with Fickian diffusion, while 0.5 < *n* < 1 indicates anomalous transport involving both diffusion and polymer relaxation, and *n* = 1 corresponds to Case II transport, generally associated with polymer relaxation or swelling-controlled transport. The precise threshold values, however, depend on the geometry and assumptions of the system and should therefore not be applied universally [269,275,276].

When applying the Korsmeyer–Peppas model, the fitting range should be considered. The model is generally intended to describe the initial portion of the release profile rather than the entire release process, and fitting is commonly restricted to the early stage of release, often up to approximately 60% cumulative release. This limitation should be considered when comparing the Korsmeyer–Peppas model with models intended to describe the complete release profile of the system and should, therefore, not be applied universally [269,274,276].

Model selection should also not depend exclusively on R^2^. Although R^2^ is useful for evaluating the correlation between experimental and fitted data, conventional R^2^ does not account for differences in model complexity and may favor models containing additional parameters. Adjusted R^2^ and information such as the Akaike Information Criterion (AIC) and the corrected AIC (AIC_C_) can be used as complementary criteria when comparing models with different numbers of parameters. These statistical criteria should be regarded as tools for model selection rather than as direct validation of a proposed physical release mechanism [70,269].

For smart wound dressings, kinetic analysis should preferably be performed separately for release data obtained under stimulated and unstimulated conditions. Comparing the fitted models, release exponents, and release parameters between the two conditions can help distinguish a genuine change in drug release. For example, a stimulus may increase the release rate without altering the underlying diffusion mechanism. In contrast, a change in the dominant transport behavior, accompanied by changes in swelling, degradation, or matrix integrity, would provide stronger evidence that the stimulus is altering the mechanism responsible for drug liberation. Kinetic modeling is most informative when combined with direct characterization of the corresponding changes in the polymer matrix [54,55,277].

A recent example was reported by De Piano et al. for pH-sensitive alginate/Carbopol hydrogel patches. Rather than relying exclusively on empirical release models, the authors combined drug release, swelling, and erosion measurements with mechanistic mathematical modeling. Their model reproduced the experimentally observed release behavior and indicated that the increased release under alkaline conditions was associated with enhanced matrix erosion, whereas release was limited under acidic conditions. This study illustrates the value of combining release kinetics with direct measurements of matrix behavior when attempting to establish stimulus specificity and the underlying release mechanism [278].

For systems containing cleavable crosslinks, such as ROS-responsive thioketal and borate ester bonds or MMP-degradable peptide crosslinks, stimulus activation may alter network integrity, mesh size, swelling, degradation, or erosion. Consequently, the response may result in increased diffusivity, enhanced matrix relaxation, network fragmentation, or increased erosion, rather than necessarily producing a single, predictable change in the kinetic model. Comparative kinetic modeling can, therefore, help identify changes in release behavior between the resting and stimulated states, but the proposed mechanism should be supported by complementary measurements of swelling, degradation, polymer mass loss, or structural changes [204,205,212,222].

This framework is also important when interpreting burst and sustained release profiles. Terms such as “burst release” and “sustained release” describe the shape or duration of a release profile but do not establish the underlying mechanism. Kinetic modeling can help determine if a release profile is correlated with diffusion-controlled transport, but it cannot independently establish whether a burst originates from rapid dissolution of surface drug, stimulus-induced erosion of the polymer network, or another process. All the possibilities should be evaluated together with complementary information on drug distribution, swelling, degradation, and matrix erosion. Similarly, a sustained release profile should not automatically be interpreted as zero-order release; it may instead result from diffusion through a hydrated polymer network or from a combination of diffusion, polymer relaxation, and degradation processes [122,269,279,280].

Overall, kinetic modeling should be used as part of a broader mechanistic characterization for assigning a release mechanism. For smart polymeric wound dressings, the strongest evidence for stimulus-controlled drug release is obtained when changes in the release profile are accompanied by corresponding changes in polymer swelling, degradation, erosion, or network structure under the stimulus. This combined approach allows a distinction between a stimulus that modifies the mechanism of drug release and one that accelerates an existing passive release process [207,281,282].

Another important aspect of polymeric smart wound dressings is a long-term stability assessment that must extend beyond conventional mechanical and chemical integrity to include retention of stimulus-responsive functionality over shelf life. Polymeric matrices may be susceptible to degradation, which can alter the responsiveness of smart functionalities such as pH-sensitive bonds, enzyme-cleavable crosslinks, or colorimetric indicators [55,267,283].

Chelminiak-Dudkiewicz et al. provided one of the few comprehensive examples, subjecting chitosan- and levan-based dressings crosslinked with dialdehyde levan to six months of climatic aging with monthly evaluation of physicochemical (FTIR, mechanical properties, swelling) and biological (antimicrobial activity, cytotoxicity) parameters. Results confirmed that the dialdehyde crosslinked network maintained structural and functional integrity. Incorporating similar protocols into smart dressing development, with explicit monitoring of stimulus response performance at each time point, is essential for clinical translation and regulatory evaluation [284].

The therapeutic scope of smart wound dressings depends not only on the responsive mechanism but also on the nature and formulation of the incorporated bioactive agent. Recent advances have expanded beyond conventional small molecule drugs to include plant-derived antioxidants, antimicrobial compounds, and growth factors encapsulated within polysaccharide matrices [10,67,98]. Do et al. reviewed how natural materials can be integrated into polysaccharide-based bioactive films for chronic wound regeneration [285]. In the context of smart systems, the formulation must ensure that the active agent remains stable within the matrix during storage while being efficiently liberated upon stimulus activation. Rizwan et al. illustrated this approach by developing mannose-decorated N-succinyl chitosan nanoparticles loaded with hesperidin and embedded within an alginate/PVA/hyaluronic acid film, achieving controlled release, hemocompatibility, and accelerated in vivo wound closure [286]. Such nanoparticle architectures are suited to smart dressings, as the nanoparticle cover can serve as an additional stimulus-responsive barrier, enabling sequential release profiles that respond to wound microenvironment changes [67].

Collectively, the methods described above provide an understanding of the structural and functional attributes, culminating in process optimization to achieve reliable, safe and effective systems.

## 6. Conclusions

This narrative review provides an examination of smart polymeric wound dressings, addressing their molecular rationale, material design and stimuli-responsive mechanisms.

Traditional wound dressings provide only passive coverage and exudate absorption, lacking the capacity to actively modulate the wound environment or respond to its dynamic biochemical changes [52,236]. This fundamental limitation has driven the development of smart polymeric wound dressings capable of responding to different signals [236].

Smart polymeric wound dressings use a multitude of endogenous and exogenous stimuli. Endogenous triggers, including pH shifts, elevated ROS, hyperglycemia, overexpressed enzymes and temperature changes, enable autonomous drug release without external intervention, with the release kinetics correlated with wound severity [53,169]. Exogenous stimuli, such as NIR light, electrical fields, magnetic fields, and ultrasound for precise therapeutic activation, can be used for targeted activation of drug release or cell migration [53,215].

The reviewed literature demonstrates that smart polymeric dressings can be engineered from a broad spectrum of natural polymers (chitosan, hyaluronic acid, alginate, cellulose, gelatin), synthetic polymers (PVA, PEG, PCL, PLGA), and their composites, each offering distinct advantages in terms of biocompatibility, mechanical properties, and degradation kinetics [87,170,236].

The main challenge of this domain consists of the implementation of smart polymeric dressings into clinical practice [52]. Several barriers contribute to this translational gap: clinical validation, regulatory complexity and different regulatory pathways across jurisdictions, maintaining batch-to-batch reproducibility, ensuring long-term stability of encapsulated bioactive agents, and achieving feasible production at industrial scale [52,287,288].

## 7. Future Perspectives for Smart Polymeric Wound Dressings

Smart polymeric wound dressings have demonstrated remarkable potential in laboratory wound-healing models, yet their implementation in daily application practice remains in its early stages. A scoping review by Probst et al., encompassing 179 studies on smart wound dressings published between 2008 and 2025, found that the majority of those were preclinical (in vitro or in vivo rodent models), with very few human investigations [283]. This gap between preclinical and clinical application defines the central challenge and opportunity for the next generation of smart polymeric wound dressings.

The wounded environment is not influenced by a single pathological signal but rather by a dynamic spectrum of pH shifts, ROS accumulation, enzymatic dysregulation, temperature fluctuations, and hyperglycemia [203,207,210,229].

The current generation of responsive wound dressings usually focuses only on one or two stimuli. In the future, the focus may be on multiple stimuli-responsive platforms that integrate more sensing mechanisms to achieve more precise therapeutic interventions [55,207,289]. This step involves temporally programmed systems that autonomously transition between therapeutic modes as the wound progresses through healing phases. For example, they may deliver antimicrobials during the inflammatory phase, anti-inflammatory agents during the transition period, and pro-angiogenic factors during proliferation, without requiring external intervention [54,204].

Progress towards such platforms is currently limited by several coexisting triggers. The contribution of an individual trigger cannot be accurately determined from a simple cumulative release curve obtained under a single stimulus. Clinically, such coupling may produce synergistic or additive effects leading to an unanticipated burst, or an antagonistic effect leading to an under-release at the moment therapy is needed [88,207].

Addressing this complexity will require a change in how responsive dressings are evaluated. Future studies should focus on factorial (or orthogonal) experimental designs. Establishing this level of mechanistic control is a prerequisite for the multi-responsive systems described above, since autonomous switching between therapeutic modes assumes that the response for each trigger is predictable, even when the others are also present [290,291]. The statistical design of experiments is already established in the formulation optimization of wound dressings and film-forming systems [171,172,249], but it has not yet been applied to the decoupling of stimulus contributions in multi-responsive dressings.

The incorporation of artificial intelligence (AI) into smart wound dressing systems may have great potential as the global trend tends to shift towards AI usage in all smart products. AI algorithms can analyze sensor data to predict healing trajectories, classify wound-healing phases, and trigger personalized, on-demand drug release from smart bandages [52,292]. Kalasin et al. designed a flexible AI-guided wearable sensor interconnected with a smart wound dressing bandage that used a neural network algorithm to achieve 94.6% accuracy in contactless healing stage recognition, classifying wounds into fast-curing, slow-curing, and no-curing regimes [293]. However, challenges related to algorithmic bias across different skin tones, a limited dataset, and evolving regulatory frameworks must be addressed before AI-assisted wound care systems can be safely deployed globally [294]. In this direction, future research may include biosensor miniaturization, omics data integration, and cloud-based platforms that enable data-driven, personalized wound care [295].

Advances in 3D bioprinting enable the fabrication of customized, patient-specific wound dressings with complex bioactive architectures that closely match individual wound geometries, overcoming the limitations of conventional dressings. Bioinks composed of natural polymers, synthetic hydrogels, extracellular matrix, and composite formulations can be precisely printed to create multilayered constructs that mimic native skin properties [131,296].

The emerging field of 4D bioprinting adds the dimension of time, incorporating stimuli-responsive materials to the evolving wound environment post-implantation. Four-dimensional printed devices can have programmed shape transformations in response to temperature, pH, moisture, or enzymatic activity, enabling autonomous wound coverage, adaptive drug release, and mechanical compliance that evolves with tissue regeneration [296,297].

The growing emphasis on environmental sustainability is driving the development of smart wound dressings based on fully biodegradable, naturally derived polymers such as chitosan, cellulose, alginate, hyaluronic acid, and gelatin. These materials offer biocompatibility and biodegradability and closely resemble the extracellular matrix [87,298].

To summarize, the future of smart polymeric wound dressings in wound care is predicted to be shaped by several technological advancements. Multi-stimuli-responsive wound dressings will enable autonomous, more phase-specific drug delivery. The integration of biosensors with wireless data transmission and AI-processed analytics will transform wound dressings into intelligent systems capable of wound monitoring and personalized intervention. However, realizing these advancements will require a fundamental shift in this field, shifting from preclinical to clinical evaluation. Future research may also prioritize the design of smart polymeric systems that maintain high therapeutic performance while minimizing the ecological footprint of wound care products.

## Figures and Tables

**Table 1:** Widely used components in polymeric wound dressings (in alphabetical order).

Polymer	Origin	Material Preparation	Crosslinking Strategy	Final Form Fabrication	Source
Alginate	Natural	Dissolution in aqueous solution	Ionic (calcium chloride, zinc chloride, barium chloride); Chemical (glutaraldehyde, citric acid)	Solvent casting, spraying	[1,48,59,60,61,62]
Carboxymethyl cellulose (CMC)	Semisynthetic	Dissolution in aqueous solution	Chemical (citric acid, epichlorohydrin)	Solvent casting, spraying	[9,63]
Chitosan (CS)	Natural	Dissolution in dilute acetic acid	Chemical (glutaraldehyde, genipin, citric acid); Ionic (sodium tripolyphosphate)	Solvent casting, spraying, electrospinning	[64,65,66,67,68,69]
Collagen	Natural	Acid or enzymatic extraction and dissolution	Chemical (1-ethyl-3-(3-dimethylaminopropyl)carbodiimide/N-hydroxysuccinimide, genipin, dialdehyde starch)	Solvent casting	[70,71,72]
Dextran	Natural	Dissolution in aqueous solution	Chemical (epichlorohydrin, periodate oxidation)	Solvent casting	[73,74]
Elastin-like polypeptides (ELP)	Synthetic *	Recombinant expression	Physical (thermal coacervation)	Solvent casting	[75]
Ethyl cellulose (EC)	Semisynthetic	Dissolution in organic solvent (ethanol, dichloromethane)	None (physical entanglement)	Solvent casting	[76]
Gelatin	Natural	Dissolution in warm aqueous solution	Chemical (glutaraldehyde, genipin, dialdehyde starch, 1-ethyl-3-(3-dimethylaminopropyl)carbodiimide/N-hydroxysuccinimide); Enzymatic (transglutaminase)	Solvent casting, electrospinning	[72,77,78]
Gellan gum	Natural	Dissolution in hot aqueous solution	Ionic (calcium chloride, potassium chloride)	Solvent casting	[79,80]
Guar gum	Natural	Dissolution in aqueous solution	Chemical (boric acid, glutaraldehyde)	Solvent casting	[73,74]
Hyaluronic acid	Natural	Dissolution in aqueous solution	Chemical (1,4-butanediol diglycidyl ether, divinyl sulfone, methacrylic anhydride); Schiff base (with oxidized polysaccharides)	Solvent casting	[1,81,82]
Hydroxyethylcellulose (HEC)	Semisynthetic	Dissolution in aqueous solution	Chemical (citric acid)	Solvent casting	[70]
Hydroxypropyl methylcellulose (HPMC)	Semisynthetic	Dissolution in aqueous solution	None (physical entanglement)	Solvent casting, spraying	[83]
Pectin	Natural	Dissolution in aqueous solution	Ionic (calcium chloride); Chemical (glutaraldehyde)	Solvent casting	[73,84]
Poly N-isopropylacrylamide (PNIPAM)	Synthetic	Free-radical polymerization	Chemical (N,N′-methylenebisacrylamide, poly(ethylene glycol)-bis(N-succinimidyl succinate))	Solvent casting	[74,85,86]
Polyacrylic acid (PAA)	Synthetic	Free-radical polymerization	Chemical (N,N′-methylenebisacrylamide); Physical (ultraviolet irradiation)	Solvent casting, electrospinning	[87,88]
Polycaprolactone (PCL)	Synthetic	Dissolution in organic solvent (chloroform, dichloromethane)	None (semi-crystalline physical network)	Solvent casting, electrospinning	[60]
Polylactic acid (PLA)	Synthetic	Dissolution in organic solvent (chloroform, dichloromethane)	None (semi-crystalline physical network)	Solvent casting, electrospinning	[89]
Polymethacrylates	Synthetic	Dissolution in organic solvent or aqueous dispersion	None (physical entanglement)	Solvent casting, spraying	[90]
Polyurethane (PU)	Synthetic	Dissolution in organic solvent (dimethylformamide, tetrahydrofuran) or aqueous dispersion	Chemical (isocyanate self-crosslinking)	Solvent casting, spraying	[91]
Polyvinyl alcohol (PVA)	Synthetic	Dissolution in hot aqueous solution	Physical (freeze–thaw cycling); Chemical (glutaraldehyde, borax, citric acid, dialdehyde cellulose)	Solvent casting, electrospinning	[92,93,94,95]
Polyvinylpyrrolidone (PVP)	Synthetic	Dissolution in aqueous or organic solvent	Physical (ultraviolet irradiation); Chemical (polyethylene glycol diacrylate)	Solvent casting, spraying	[96,97,98]
Pullulan	Natural	Dissolution in aqueous solution	Physical (alkaline gelation with chitosan)	Solvent casting	[67,99,100]
Silk fibroin	Natural	Degumming, dissolution in lithium bromide, dialysis	Physical (methanol or ethanol induced β-sheet formation); Chemical (genipin); Enzymatic (horseradish peroxidase)	Solvent casting	[101,102,103]

* This polymer was obtained through a bioengineering process.

**Table 2:** A summary of characterization methods for Smart Polymeric Wound Dressings.

Method	Property Measured	Relevance	Sources
Structural and Morphological Characterization			
SEM (Scanning Electron Microscopy)	Surface and sectional morphology, porosity	Evaluates homogeneity, pore architecture and drug or nanoparticle distribution.	[238,239]
TEM (Transmission Electron Microscopy)	Nanoscale internal structure, nanoparticle size and dispersion	Visualizes responsive nanoparticles, like nanozymes, metal nanoparticles within the polymeric matrix; confirms particle morphology critical for stimulus-responsive function	[236,238]
AFM (Atomic Force Microscopy)	Surface roughness (at nanoscale), topography	Quantifies nanoscale surface roughness and mechanical heterogeneity. Assesses cell interaction surfaces.	[240,241]
XRD (X-Ray Diffraction)	Crystallinity and phase structure	Distinguishes between amorphous and crystalline drug and polymer states, both affecting release profiles	[242]
Chemical and Compositional Characterization			
FTIR (Fourier-Transform Infrared Spectroscopy)	Detects chemical bonds, functional groups and molecular interactions	Confirms polymer–drug compatibility, identifies hydrogen bonding, verifies successful formation of stimulus-responsive bonds (e.g., borate ester, Schiff base), and detects chemical changes after stimuli exposure	[70,238]
HPLC (High-Performance Liquid Chromatography)	Drug content and release quantification, degradation products	Quantifies drug loading efficiency and release profiles. Identifies and quantifies degradation byproducts formed during ROS- or enzyme-triggered release	[240,241]
UV-Vis Spectrophotometry	Drug concentration, colorimetric response	Monitors drug release kinetics in dissolution studies and quantifies colorimetric responsive dye changes in smart dressings	[237,241]
LC-MS/MS (Liquid Chromatography–Tandem Mass Spectrometry)	Structural identification and quantification of degradation products	Identifies fragments released by stimulus-triggered bond degradation essential for toxicological evaluation where degradation products differ structurally from the parent polymer	[243,244,245]
Thermal Characterization			
DSC (Differential Scanning Calorimetry)	Glass transition temperature, melting point, crystallization temperature	Determines thermal stability and physical state of polymer and drug (crystalline/amorphous)	[238,239]
TGA (Thermogravimetric Analysis)	Thermal degradation/weight loss	Assesses decomposition profiles and thermal stability of polymer networks	[236,237]
Mechanical Characterization			
Elasticity	Ability to recover original shape after deformation	Ensures the dressing can flex with body movement and return to its original form.	[236,238]
Folding endurance	Number of repeated folds before cracking or breaking	Assesses durability and flexibility for application over joints and curved body surfaces; ensures long-term mechanical integrity during wear	[238,239]
Breaking Strain	Maximum deformation before material failure	Determines the elongation limit of the dressing under stress	[246,247]
Physicochemical Characterization			
Drying rate	Rate of solvent evaporation and film formation	Influences final thickness, homogeneity and manufacturing reproducibility	[248]
pH	Determining acidity or alkalinity	Ensures product-to-skin compatibility and chemical stability of ingredients	[247]
Viscosity	Flow behavior of polymeric solution or gel	Controls spreadability and castability	[247,249]
Density	Mass per unit volume of a material	Reflects polymer packing and structural integrity. Affects final weight.	[249,250,251]
Surface tension and contact angle	Surface wettability	Indicates hydrophilic/hydrophobic nature, crucial for fluid interaction and cell attachment	[70,252]
Moisture content	Assesses the residual water content in a wound dressing	Influences mechanical properties, drug stability and microbiological susceptibility.	[253]
Thickness	Thickness uniformity	Directly affects drug loading, release kinetics and mechanical properties	[70,254]
Functional Performance Characterization			
Swelling	Hydration behavior	Predicts how dressings absorb wound exudate and influence drug diffusion	[70]
Mucoadhesion	Adhesive strength to (wet) surfaces	Measures the ability to adhere to the wound site and improves localized drug delivery	[235]
Tackiness	Initial adhesion to skin	Determines immediate contact adhesion upon application, influencing dressing application time for some patients	[70]
Vapor permeability/Gas permeability/Moisture permeability	Water vapor and gas transmission rates, measured in g/m^2^/day	Indicates breathability and moisture management capability of the dressing	[108,255]
Transparency	The property of allowing light to pass through.	Allows visual wound monitoring without removing the wound dressing	[49,256]
Pharmaceutical and Drug Delivery Characterization			
Dosing uniformity/content per dose	Homogeneity of drug distribution per unit dose	Ensures uniform drug distribution, critical for consistent therapeutic effect across the wound surface	[235]
Drug release and kinetics modeling	Cumulative drug release over time and fitting of data to zero-order, first-order, Higuchi and Korsmeyer–Peppas models	Characterizes the rate and mechanism of drug liberation from the polymeric matrix and identifies how much the release is controlled by diffusion or erosion and how much the stimulus alters the release mechanism	[70,122,131,235]
Stimuli Responsive (Smart) Functionality			
Stimulus-response dose dependence	Output magnitude (drug released, color change, current, swelling) as a function of stimulus intensity	Establishes the calibration curve, sensitivity and working range, distinguishes genuine responsiveness from passive release	[52,54,55]
Response time	Time to reach a defined fraction of steady output after stimulus action	Determines if the system acts within optimal time of stimulus action	[181,198]
Reversibility and repeated response cycles	Output amplitude over consecutive stimulus on/off cycles (amplitude loss across cycles)	Separates reversible sensing functions, which must be reusable over the wear period, from irreversible triggers and assesses lifetime	[174,181]
Hysteresis	Difference in output between ascending and descending stimulus sweeps	Quantifies the error introduced in the system by fluctuating wound conditions	[257]
Signal stability and baseline drift	Baseline shift per unit time during continuous operation (>48 h)	Defines the maximum wear time of continuously operating sensors	[182,184]
Microbiological Performance			
Antimicrobial efficacy	Log_10_ colony-forming units reduction or zone of inhibition	Demonstrates active antimicrobial function	[98,105]
Repeated antimicrobial efficacy	Tests efficacy after multiple inoculations of the medium	Reflects prolonged wear time rather than a single exposure	[258]
Microbial barrier	Bacterial penetration through the intact dressing	Confirms protection against exogenous contamination	[40,259,260]
Biocompatibility			
Cytotoxicity	Cell viability upon exposure to product	Evaluates if the components are toxic to mammalian cells and is typically assessed through ISO-compliant assays such as MTT	[173,234,239]
Cytotoxicity after degradation	Cell viability upon exposure to extracts of isolated degraded material	Responsive chemistries release defined fragments whose toxicity differs from that of the intact dressing	[261]
Hemocompatibility	Percentage hemolysis of erythrocytes after contact	Required for materials contacting bleeding tissue	[262,263]
Skin irritation test	Dermal tolerance and allergy potential	Assesses local inflammatory or allergic response upon skin contact; can be evaluated via in vitro 3D skin models or in vivo patch testing	[264]
Biodegradation	Mass loss and morphological breakdown in simulated wound fluid or enzyme-containing media	Determines whether the dressing must be removed or can resorb in situ	[60,87,223,263]
Stability			
Visual uniformity	Macroscopic homogeneity	Confirms absence of visible defects (bubbles, cracks, phase separation, color irregularities) across the dressing surface	[70,249,265]
Stability	Physicochemical and functional integrity during shelf life	Evaluates product aging to confirm that mechanical, chemical, and drug release properties are maintained throughout the shelf life	[266,267]
Content uniformity over time	Consistency of drug content per unit area across the dressing, monitored at successive storage time points	Confirms that the drug remains homogeneously distributed and that total drug content does not decline through degradation, migration, or crystallization during shelf life; deviation over time signals chemical instability or phase separation that would compromise dose reproducibility	[267,268]

## Data Availability

The original contributions presented in this study are included in the article. Further inquiries can be directed to the corresponding author.
